# Advancing Compliance with HIPAA and GDPR in Healthcare: A Blockchain-Based Strategy for Secure Data Exchange in Clinical Research Involving Private Health Information

**DOI:** 10.3390/healthcare13202594

**Published:** 2025-10-15

**Authors:** Sabri Barbaria, Abderrazak Jemai, Halil İbrahim Ceylan, Raul Ioan Muntean, Ismail Dergaa, Hanene Boussi Rahmouni

**Affiliations:** 1 Laboratory of Biophysics and Medical Technologies, LR13ES07 (BTM), Higher Institute of Medical Technologies of Tunis (ISTMT), University of Tunis El Manar, Tunis 1080, Tunisia; sabri.barbaria@istmt.utm.tn (S.B.); hanene.boussi@istmt.utm.tn (H.B.R.); 2 SERCOM-Lab, Tunisia Polytechnic School, INSAT, Carthage University, Tunis 1080, Tunisia; abderrazak.jemai@insat.rnu.tn; 3 Physical Education and Sports Teaching Department, Faculty of Sports Sciences, Ataturk University, 25240 Erzurum, Türkiye; 4 Department of Physical Education and Sport, Faculty of Law and Social Sciences, University “1 Decembrie 10 1918” of Alba Iulia, 510009 Alba Iulia, Romania; 5 High Institute of Sport and Physical Education of Ksar Said, University of Manouba, Manouba 2010, Tunisia; phd.dergaa@gmail.com; 6 The Computer Science Research Centre, The University of the West of England, Bristol BS16 1QY, UK

**Keywords:** attribute-based access control, blockchain technology, cryptographic protocols, data interoperability, GDPR compliance, healthcare informatics, HIPAA regulations, Hyperledger Fabric, privacy preservation, smart contracts

## Abstract

**Background:** Healthcare data interoperability faces significant barriers, including regulatory compliance complexities, institutional trust deficits, and technical integration challenges. Current centralized architectures demonstrate inadequate mechanisms for balancing data accessibility requirements with patient privacy protection, as mandated by HIPAA and GDPR frameworks. Traditional compliance approaches rely on manual policy implementation and periodic auditing, which are insufficient for dynamic, multi-organizational healthcare data-sharing scenarios. **Objective:** This study develops and proposes a blockchain-based healthcare data management framework that leverages Hyperledger Fabric, IPFS, and the HL7 FHIR standard and incorporates automated regulatory compliance mechanisms via smart contract implementation to meet HIPAA and GDPR requirements. It assesses the theoretical system architecture, security characteristics, and scalability considerations. **Methods:** We developed a permissioned blockchain architecture that employs smart contracts for privacy policy enforcement and for patient consent management. The proposed system incorporates multiple certification authorities for patients, hospitals, and research facilities. Architectural evaluation uses theoretical modeling and system design analysis to assess a system’s security, compliance, and scalability. **Results:** The proposed framework demonstrated enhanced security through decentralized control mechanisms and cryptographic protection protocols. Smart contract-based compliance verification can automate routine regulatory tasks while maintaining human oversight in complex scenarios. The architecture supports multi-organizational collaboration with attribute-based access control and comprehensive audit-trail capabilities. **Conclusions:** Blockchain-based healthcare data-sharing systems provide enhanced security and decentralized control compared with traditional architectures. The proposed framework offers a promising solution for automating regulatory compliance. However, implementation considerations—including organizational readiness, technical complexity, and scalability requirements—must be addressed for practical deployment in healthcare settings.

## 1. Introduction

Healthcare digitization has created unprecedented opportunities for collaborative medicine while simultaneously amplifying privacy protection challenges across institutional boundaries [1,2,3]. The global healthcare information technology market exceeded $350 billion in 2022, reflecting the widespread recognition of the importance of digital infrastructure for coordinated patient care delivery [4,5]. Modern healthcare organizations are increasingly recognizing that traditional, siloed data management approaches significantly limit their capacity to participate in collaborative care networks, conduct meaningful research initiatives, and achieve optimal patient outcomes through coordinated treatment protocols [6,7,8]. However, the transition from isolated information systems to integrated collaborative platforms remains constrained by technical interoperability barriers, regulatory compliance complexity, and fundamental trust deficits among healthcare institutions [9,10].

The regulatory landscape governing healthcare data sharing is substantially complex, with frameworks such as the Health Insurance Portability and Accountability Act (HIPAA) in the United States and the General Data Protection Regulation (GDPR) in the European Union mandating specific technical safeguards, administrative procedures, and physical security measures [11,12]. These regulations require healthcare organizations to implement comprehensive privacy protection mechanisms when collecting, processing, storing, or sharing protected health information across organizational boundaries [13,14,15]. Contemporary compliance approaches typically rely on manual policy implementation, periodic audit procedures, and reactive security incident responses, which are proving increasingly inadequate for addressing the dynamic requirements of modern multi-organizational healthcare data-sharing scenarios [16,17]. The interpretation and practical implementation of complex regulatory requirements vary significantly across healthcare organizations, frequently resulting in overly conservative data-sharing policies that inadvertently limit beneficial healthcare research collaborations and clinical care coordination initiatives [18,19,20].

Current centralized healthcare information systems exhibit fundamental architectural limitations that create significant barriers to secure, scalable, and efficient data sharing across organizational boundaries [21,22]. These systems characteristically implement single-point-of-control architectures that inherently create security vulnerabilities, lack comprehensive, immutable audit-trail capabilities, and provide insufficient mechanisms for granular patient consent management [23,24]. The centralized architecture model necessitates individual bilateral trust relationships between participating organizations, complex legal data-sharing agreements, and technically compatible infrastructure implementations, creating substantial administrative overhead and technical barriers to collaborative initiatives [25,26]. Furthermore, centralized systems typically struggle to provide real-time compliance verification, transparent audit mechanisms, and flexible consent management frameworks that can accommodate evolving patient preferences and changing regulatory requirements [27,28].

Blockchain technology, best known for its use in cryptocurrencies such as Bitcoin, shows enormous promise in healthcare [29]. It utilizes a decentralized ledger to maintain tamper-resistant transaction records. Each new piece of data was connected to the previous data to ensure data integrity. This entails building an auditable and immutable medical record for patients, thereby encouraging trust, privacy, and accountability among patients, providers, and institutions.

Blockchain enhances healthcare by addressing various challenges stemming from its inherent characteristics. The intrinsic trust-building aspect of blockchains overcomes the weaknesses of centralized systems, promoting data integrity and security through distributed consensus mechanisms [30,31,32]. Anonymity and private transaction capabilities supported by specific blockchain systems effectively safeguard patient privacy and protect resource owners from unauthorized data exposure [33,34,35]. Furthermore, the ability of blockchain to maintain data-sharing policies without requiring modifications to existing IoT devices aligns with the lightweight nature required by healthcare systems [36,37].

The scalability potential of blockchain technology enables the accommodation of numerous resource owners through smart contract implementation, potentially alleviating the scalability difficulties that plague traditional systems [38,39]. Permissioned blockchain implementations can achieve the necessary transaction throughput while maintaining robust data security protocols, which are essential for healthcare applications [40,41,42]. The inherent transparency of blockchain systems can significantly reduce fraud and corruption in healthcare delivery, fostering enhanced accountability between healthcare practitioners and patients [43,44]. Additionally, the decentralized nature of the blockchain architecture ensures uninterrupted access to essential medical data and maintains system availability even during localized system failures or infrastructure disruptions [45,46].

Based on a comprehensive analysis of the identified research gaps in healthcare data-sharing methodologies, particularly the documented limitations of centralized system architectures and substantial challenges associated with automated regulatory compliance implementation, this study proposes a blockchain-based framework capable of addressing technical performance requirements and complex regulatory compliance needs for secure healthcare data sharing across multiple organizational boundaries.

While prior research has examined blockchain applications for healthcare data sharing and regulatory compliance separately [11,47,48], a notable research gap remains: a dedicated architectural strategy that systematically integrates and automates compliance with both HIPAA and GDPR within a unified, multi-organizational blockchain framework. This study seeks to address this gap by proposing a comprehensive framework that incorporates regulatory compliance automation directly into a secure data-sharing infrastructure, advancing beyond general discussions to a concrete, implementable design.

## 2. Background and Related Work

Building on the challenges identified in current healthcare data-sharing systems, this section examines the evolution of healthcare information technologies. This study explores how blockchain-based solutions have emerged to address these persistent limitations. The analysis progressed from traditional healthcare information systems to contemporary blockchain applications, establishing a theoretical foundation for the proposed architectural framework.

The management and sharing of healthcare data for research purposes present significant challenges in terms of security, privacy, and regulatory compliance. Traditional centralized systems often struggle to balance the need for data accessibility with the imperative of protecting sensitive patient information. In recent years, blockchain technology has emerged as a promising solution for addressing these challenges in healthcare data management. This comprehensive examination explores how blockchain technology, particularly Hyperledger Fabric, can be leveraged to create a secure, transparent, and compliant system for managing protected health information (PHI) while facilitating essential research collaborations across institutional boundaries.

### 2.1. Healthcare Information Systems and Healthcare 4.0

A healthcare information system (HIS) encompasses a collection of concrete and abstract resources that facilitate the handling of clinical data, enabling medical staff to improve patient care management [49]. Many studies have been conducted to develop and improve the quality of such systems, as they have a crucial impact on our lives by enabling the accurate and understandable availability of healthcare data.

Recent research has explored the motivations behind cyberattacks and the challenges they pose, with a particular focus on phishing attacks in the healthcare sector [50]. Preventive measures to combat this threat emphasize the importance of a comprehensive approach to the problem. By adopting these strategies, healthcare organizations can strengthen their defenses, safeguard patient information, maintain the integrity of medical systems, and cultivate a cybersecurity culture in the digital age.

A blockchain-inspired architecture for secure and reliable data exchange in the Cyber-Physical Healthcare Industry 4.0 was proposed, utilizing BigchainDB, Tendermint, Inter-Planetary File System (IPFS), MongoDB, and AES encryption algorithms to enhance healthcare 4.0 [51]. This system introduces a blockchain-enabled healthcare architecture that enables secure access to and management of records between doctors and patients, prioritizes patient control, and empowers individuals to have complete authority over their data. Utilizing blockchain technology ensures the security and privacy of the data.

### 2.2. Blockchain and Smart Contract Applications

The evolution from traditional healthcare information systems to more sophisticated blockchain-based solutions represents a natural progression in addressing the limitations identified in centralized architectures. Since the emergence of blockchain technology and its introduction into Bitcoin by Nakamoto [29], numerous studies have been conducted to develop distributed techniques across multiple domains, including finance, industry, supply chains, and healthcare. Since 2015, there has been an increase in scientific and academic publications on blockchain technology in healthcare, driven by the enormous potential of blockchain to transform the current healthcare model into a safer, more robust, and distributed system [52].

Smart contracts are among the most critical components of blockchain systems, implementing self-executing contracts in healthcare by managing processes across finance, staff, patient care, and legal matters. The implementation of smart contracts can enhance efficiency, increase compliance, and introduce a layer of trust to the ecosystem [53]. Recent research has proposed architectures for healthcare blockchain-based smart contracts that prioritize performance while ensuring the security of private medical data [54]. These innovative contract implementations demonstrate the potential to automate complex healthcare workflows while ensuring adherence to security and compliance requirements essential to safeguarding patient data.

### 2.3. Compliance Frameworks

The critical importance of regulatory compliance in healthcare data sharing requires sophisticated frameworks that address the complex requirements of both HIPAA and GDPR regulations. To achieve the highest level of maturity in healthcare systems, compliance protocols must be implemented that address not only technical security requirements but also legal and procedural mandates [47]. Many studies have highlighted the importance of regulatory compliance and proposed algorithms and system designs to ensure it. However, significant gaps remain in automated compliance verification systems that can operate effectively across multiple jurisdictions.

A comparison between the GDPR of the European Union and the HIPAA of the United States concerning the collection, use, and protection of health information (PHI) reveals significant differences in scope, consent requirements, and enforcement mechanisms. The GDPR governs the use of personal data and applies to all data concerning individuals within its jurisdiction, demonstrating its comprehensive scope [55]. Regarding covered entities, the GDPR expanded previous EU regulations to include organizations outside the EU that process personal data of EU-based individuals. However, HIPAA has a narrower focus, regulating entities involved in the electronic transmission of PHI and their business associates [15,56].

Recent research has proposed ontology-based compliance audit frameworks for medical data sharing in accordance with GDPR requirements, enabling privacy auditors to examine past data-processing events and verify compliance with legal privacy requirements [48]. Formal modeling and analysis of GDPR compliance in IoT healthcare systems have also been developed, describing various GDPR components and illustrating the results for monitored patient scenarios [57]. However, these existing approaches primarily focus on post hoc compliance verification rather than on proactive, real-time compliance automation integrated into the data-sharing infrastructure. This limitation highlights the need for architectural solutions that integrate compliance verification directly into data exchange protocols, which form the foundation of the proposed blockchain-based framework presented in the following section.

## 3. Proposed Architecture

Drawing from the identified limitations of existing healthcare information systems and the gaps in current compliance automation approaches, this section presents a comprehensive blockchain-based framework designed to address the specific challenges of multi-organizational healthcare data sharing while ensuring automated regulatory compliance. The proposed architecture integrates the distributed security benefits of blockchain technology with sophisticated smart contract-based compliance verification, creating a system that can facilitate secure data exchange across institutional boundaries while maintaining strict adherence to HIPAA and GDPR requirements. The architectural design progresses from fundamental compliance requirements to technical implementation details and system-level integration considerations, thereby providing a comprehensive solution framework for healthcare data-sharing challenges.

### 3.1. Compliance Approach

To create a compliance framework, we must extract responsibilities from HIPAA and GDPR regulations and translate them into algorithms that can be understood as smart contracts for implementation on blockchain networks. Our study focused on HIPAA research components, including the use of protected health information (PHI) and the obtaining of patient consent.

Our architectural framework is designed to meet the requirements of the HIPAA and GDPR. However, the initial smart contract logic and pseudocode presented in this study primarily address HIPAA provisions concerning the research use of Protected Health Information (PHI). This focus was selected as a foundational step because of HIPAA’s well-defined permissible use cases and authorization requirements, which are particularly amenable to algorithmic translation. The modular design of the framework facilitates the future integration of additional smart contracts that encapsulate the GDPR’s more context-dependent provisions.

Table 1 summarizes the questions related to HIPAA requirements for using, generating, or communicating PHI. These questions form the foundation for implementing smart contract logic. HIPAA enables the use of PHI for research purposes by ensuring compliance with requirements and participation in research projects approved by an Institutional Review Board (IRB). PHI is necessary for research projects in two aspects: when studies require medical record review as the sole source of research information, and when studies involve creating new medical records as part of research examining novel medications or technologies.

Table 2: The decision logic framework governing HIPAA authorization for the use of Protected Health Information (PHI) is illustrated in this section. This framework formalizes the evaluation process using a structured set of conditional statements that determine whether authorization is granted or denied. Each decision path is based on a combination of initial consent indicators (Q001–Q002), path conditions (Q003), and a series of compliance criteria (Q004–Q011) that reflect the HIPAA waiver requirements. When direct consent or authorization is available, the use of PHI is permitted immediately; otherwise, authorization may proceed only under a validated HIPAA Waiver, provided that all regulatory safeguards, such as privacy impact assessments, data de-identification, and access rights, are satisfied.

### 3.2. Hyperledger Fabric Implementation

Building on the foundational compliance requirements established in the previous section, the technical implementation of the proposed framework utilizes Hyperledger Fabric as its core blockchain platform. This choice was made for its permissioned network capabilities and enterprise-grade features, which are essential for healthcare applications. We developed a healthcare blockchain model inspired by the Hyperledger Fabric framework, designed for deployment using containerization technology that provides the scalability and isolation necessary for multi-organizational healthcare environments.

The system architecture treats containers as separate host machines to observe the asset exchange requirements between network participants, enabling a realistic simulation of a distributed organizational infrastructure while maintaining a controlled testing environment. The fabric-ca-client binary must be present on each host machine that requires cryptographic content acquisition, ensuring secure key management and certificate distribution across all participating healthcare organizations.

The Certificate Authority (CA) infrastructure is a critical component of the security architecture, issuing TLS certificates that are essential for securing communication between different processes within the network. Each participating organization maintains its own CA to issue certificates to peers and clients, generate organizational identities, and assign public–private key pairs that establish cryptographic trust. All nodes and applications use these keys to sign and validate activities, with any CA-signed identity recognized by other network members, according to predefined trust policies. This distributed certificate management approach enables healthcare organizations to retain control over their identity infrastructure while participating in collaborative data-sharing networks.

### 3.3. System Architecture Design

**Figure 1:**
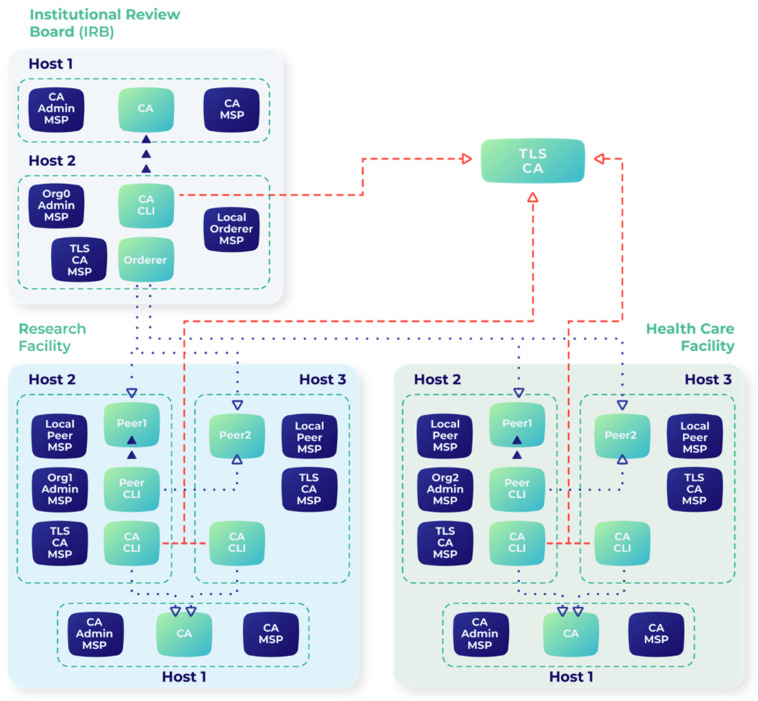
Low-level architecture of the Docker container of the proposed blockchain approach.

The proposed system implements a secure, efficient procedure for recording research facility registration information on a blockchain ledger. Figure 1 illustrates the comprehensive deployment of Docker containers in our implementation, treating containers as separate host machines to observe the asset exchange requirements between network participants. The architecture comprises multiple components, including peer nodes, ordering services, and certificate authorities.

**Figure 2:**
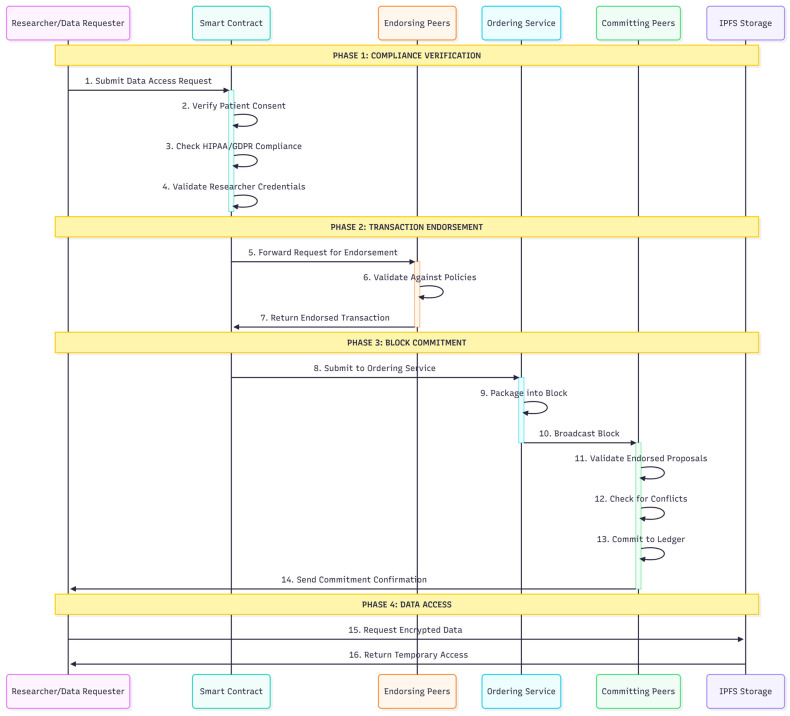
Data Access Request Process.

Figure 2 illustrates the data access request process, which demonstrates how research facilities submit registration forms containing identity information while generating cryptographic key pairs. The registration process involves facility identity verification through cryptographic procedures, the generation of a public–private key pair, and the issuance of an electronic certificate by local Certificate Authorities. Proposal endorsement involves multiple peer verifications before recording on the blockchain to ensure thorough validation and compliance with predefined policies.

**Figure 3:**
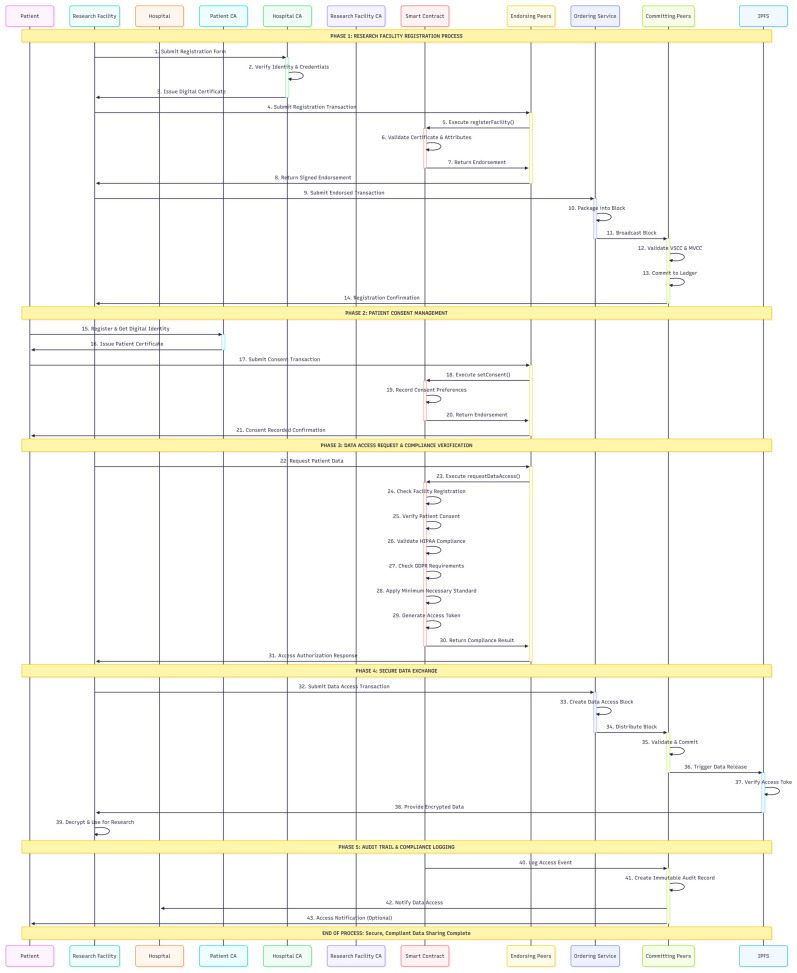
Detailed steps and interactions between various components involved in the registration and data access processes.

Figure 3 outlines the comprehensive steps and interactions among the components involved in the registration and data access processes, ensuring a robust, secure system for managing research facility information and data transactions in the blockchain ledger. Data access requests undergo compliance checks by the data providers, followed by peer network endorsement. Data encryption uses symmetric keys, which are further encrypted with public-key pairs to ensure confidentiality during transmission and storage.

To illustrate the practical application of this architecture, we considered a multicenter clinical trial in which Hospital A needed to share specific patient data with Research Facility B. The process unfolds as follows: First, the patient provides consent via a smart contract, which is immutably recorded on the ledger. When a researcher requests data, the smart contract automatically verifies the credentials and patient consent against the predefined research protocol. Only then is a temporary encrypted link to the data (stored on IPFS) provided, with the entire access event logged on the blockchain for permanent audit. This demonstrates how an abstract blockchain architecture can be translated into tangible and secure healthcare data-sharing workflows.

### 3.4. Performance and Scalability Considerations

**Figure 4:**
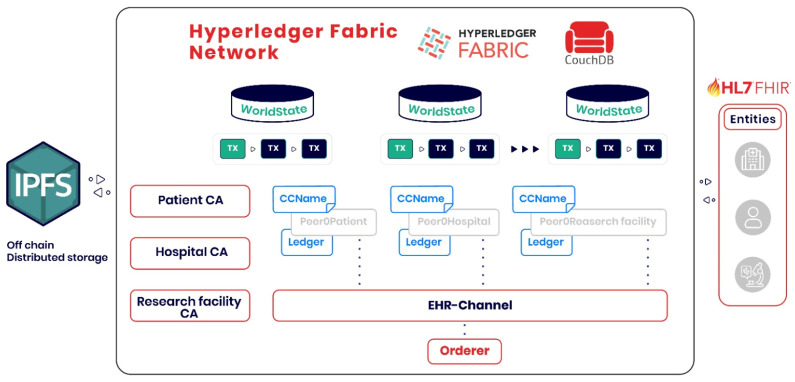
Schematic Representation of a Blockchain-based Healthcare Data Management System.

The proposed architecture demonstrates the potential to handle substantial transaction loads while maintaining security and compliance standards. Figure 4 presents a schematic of the blockchain-based healthcare data management system, illustrating an architecture that uses Hyperledger Fabric and IPFS for distributed storage. The system features three main certification authorities (CAs): the Patient CA, the Hospital CA, and the Research Facility CA, which authenticate and authorize the respective entities within the network.

**Figure 5:**
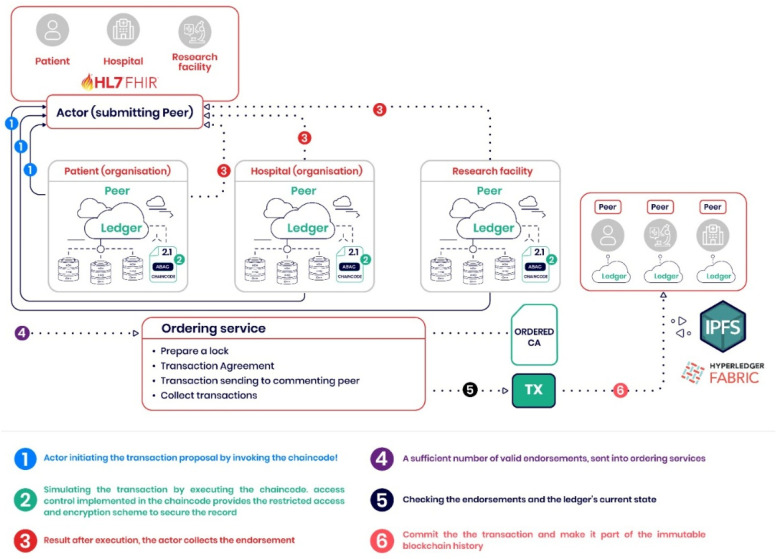
Transaction Flow in a Blockchain-based Healthcare Data Management System.

Figure 5 outlines the transaction flow within the blockchain-based healthcare data management system, demonstrating the comprehensive process from transaction initiation to final commitment. The theoretical analysis suggests that the system can process transaction rates suitable for clinical environments, with scalability characteristics that support multi-organizational healthcare collaboration.

The performance characteristics vary with network configuration complexity, with single-organization deployments showing higher throughput than multi-organizational networks. The endorsement policy requirements that mandate transaction approval from multiple peer nodes contribute to processing overhead but ensure security and compliance verification.

Resource utilization analysis indicates that computational requirements scale with network participation levels, necessitating the consideration of infrastructure investment and operational costs. The linear relationship between organizational complexity and resource consumption suggests that proportional scaling requirements are necessary for larger healthcare collaborations.

#### Proposed Evaluation Methodology

To transition from theoretical design to empirical validation, the following evaluation methodology is proposed for future implementation of this framework:

1. Performance Benchmarking: The framework will be implemented as a prototype on Hyperledger Fabric. Performance will be measured using the Hyperledger Caliper benchmarking tool to collect key metrics under varying loads [58]:
  - Transaction Throughput (TPS): Measured for core operations, including patient consent recording, data access requests, and compliance verification.
  - Transaction Latency: The time from transaction submission to ledger commitment, which is divided into endorsement, ordering, and validation phases.
  - System Resource Consumption: CPU and memory usage of peer nodes and ordering services will be monitored to assess infrastructure requirements.
2. Scalability Analysis: The network will be tested in multiple configurations, starting with a basic 2-organization setup and scaling up to 10+ organizations. This will assess the impact of increased network size and complexity on the performance metrics listed above [59].
3. Security and Compliance Verification:
  - Formal Verification: The smart contract logic for HIPAA authorization (Table 2) will be formally verified using tools such as KEVM or Mythril to ensure that the code correctly implements the specified policy [60,61].
  - Penetration Testing: The network architecture and APIs will be subjected to controlled penetration testing to identify potential vulnerabilities in access control and data flows [62].
4. Comparative Analysis: The performance and features of the proposed framework will be compared against:
  - A baseline centralized architecture with manual compliance check.
  - Other blockchain-based healthcare data management systems from the literature were compared based on their reported performance metrics.

This structured methodology provides a roadmap for quantitatively validating the theoretical advantages of this conceptual design.

## 4. Security and Compliance Analysis

Having established the architectural foundation and technical implementation details of the proposed blockchain framework, this section examines the security mechanisms and compliance capabilities that distinguish the system from traditional centralized approaches. The analysis progressed through cryptographic security implementations, automated regulatory compliance verification, and interoperability considerations, demonstrating how the proposed architecture addresses the fundamental challenges identified in current healthcare data-sharing systems. Security evaluation encompasses both technical security measures and regulatory compliance automation, providing a comprehensive assessment of the framework’s ability to protect sensitive healthcare information while facilitating essential research collaborations.

### 4.1. Cryptographic Security Implementation

A comprehensive cryptographic security implementation demonstrates robust protection characteristics through multiple layers of security. Digital signature verification systems ensure transaction authenticity, and encryption algorithms maintain data confidentiality during storage and transmission. Patient identity protection mechanisms prevent unauthorized access through attribute-based access-control systems.

The cryptographic erasure protocol offers a practical solution to the GDPR’s right-to-erasure requirements by rendering the primary off-chain data on IPFS inaccessible through patient-controlled key management. However, it is acknowledged that specific transaction metadata on an immutable blockchain ledger may persist. To fully address GDPR, a comprehensive strategy is required, potentially involving the initial use of advanced cryptographic techniques, such as zero-knowledge proofs, to minimize the storage of personal identifiers on-chain. This ensures that persistent metadata cannot be linked to an identifiable individual after erasure. While patient-controlled key management empowers individuals, it introduces practical challenges, including the risk of key loss. To mitigate this, a strategy employing threshold cryptography (Shamir’s Secret Sharing) is proposed. A patient’s private key would be split into shares, with parts held by the patient and designated, trusted entities (e.g., their primary care provider and a designated family member). This allows key recovery without vesting full control in a single institution, thereby enhancing robustness without compromising the decentralized trust model.

### 4.2. Regulatory Compliance Automation

The integration of automated compliance verification within blockchain architecture represents a significant advancement over traditional manual compliance approaches that rely on periodic auditing and reactive incident responses. The automated regulatory compliance verification system demonstrated the potential to process routine compliance tasks while maintaining the necessary human oversight for complex legal interpretation. Smart contract-based rule evaluation systems implement HIPAA and GDPR requirements by verifying them through automated assessment protocols that operate in real time during data access requests and sharing transactions.

HIPAA compliance verification includes procedures that automatically verify the legitimacy and currency of patient authorizations before granting access to data. The system implements granular access control enforcement mechanisms that ensure that only authorized personnel can access specific data elements, adhering to the minimum necessary standard mandated by HIPAA regulations. A comprehensive audit trail generation creates immutable records of all data access events, providing the documentation required for regulatory compliance verification and potential investigations.

GDPR compliance assessment protocols include sophisticated data subject consent management capabilities that track and verify the legal basis for data-processing activities across multiple organizational boundaries. The system implements technical solutions for the right to erasure requirements through cryptographic mechanisms that can render data inaccessible, while maintaining the integrity of the blockchain ledger. Automated data-processing lawfulness verification procedures ensure that all data-sharing activities comply with the GDPR requirements for data controller–processor relationships. Cross-border transfer compliance monitoring provides continuous verification that international data-sharing activities meet the adequacy requirements established by the European data protection authorities.

The hybrid approach, which combines automated routine task processing with expert human oversight, represents a realistic implementation strategy that acknowledges the inherent limitations of algorithmic legal interpretation while maximizing the efficiency gains available through automation technologies.

To illustrate the translation of nuanced provisions, consider the ‘minimum necessary’ standard under HIPAA. This is implemented by defining data attributes (e.g., dataCategory: [demographics, lab_results, mental_health]) and binding them to specific research purposes, as consented to by the patient (recorded on-chain). A smart contract enforcing a data access request cross-references these attributes. For example, a researcher authorized for a ‘cardiology study’ might be automatically granted access only to lab results related to cardiac function. In contrast, access to mental health records would be denied as non-essential, thus algorithmically enforcing the ‘minimum necessary’ principle.

### 4.3. Interoperability and Integration

The HL7 FHIR standard implementation is compatible with the existing healthcare data format requirements, supporting seamless integration with contemporary healthcare information systems. The IPFS integration for off-chain distributed storage demonstrates reliable performance characteristics in healthcare document management.

To ensure interoperability with legacy healthcare systems that lack native FHIR support, the framework supports FHIR converter services or adapter APIs. These components are responsible for mapping and transforming legacy data formats (e.g., HL7v2, CDA) into standardized FHIR resources before they are processed or stored by the blockchain system, enabling a practical and incremental adoption path [63].

External healthcare system integration capabilities enable connectivity with existing healthcare information systems. However, the complexity of integration requires specialized knowledge of blockchain technology and technical implementation expertise. User training needs include education on blockchain technology for administrative personnel and training on system interface interaction for healthcare professionals.

## 5. Discussion

This comprehensive analysis revealed the significant capabilities and considerations that healthcare organizations must evaluate before implementing blockchain-based data-sharing systems in clinical environments.

### 5.1. Architectural Advantages and Limitations

The proposed blockchain-based framework addresses the fundamental limitations of centralized healthcare information systems by providing decentralized control mechanisms, an immutable audit trail, and granular patient consent management. The architecture eliminates single points of failure while enabling transparent audit mechanisms and flexible consent management frameworks.

However, the complexity of multi-organizational blockchain networks introduces performance considerations that must be balanced against the benefits of security and compliance [64]. The endorsement requirements necessary for transaction validation create a processing overhead that scales with the level of network organizational participation [65,66]. Although the permissioned nature of Hyperledger Fabric avoids the high energy consumption associated with proof-of-work blockchains, such as Bitcoin, the computational overhead of cryptographic operations remains a valid consideration for large-scale deployments [67].

Additionally, the distributed nature of blockchain systems, while enhancing security and resilience, may raise skepticism among healthcare organizations accustomed to centralized control models [68]. Although beneficial for audit trails, the irreversible nature of blockchain transactions requires careful implementation to ensure system flexibility and error-correction capabilities without compromising security principles [69].

To provide quantitative context for the scalability discussion, a theoretical performance model was developed based on established Hyperledger Fabric characteristics [59,61]. The model projects that a network with 10 organizations could sustain a transaction throughput of 150–300 TPS, suitable for regional research consortia. Scaling to 50 organizations would yield a throughput of 80–180 TPS, enabling statewide collaborations. Meanwhile, a large consortium of 100 organizations could maintain a throughput of 30–100 TPS, sufficient for international research data aggregation. These projections assume a default endorsement policy and are intended to provide a numerical framework for feasibility assessment, with the understanding that actual performance will be empirically validated as outlined in our evaluation methodology.

### 5.2. Regulatory Compliance Considerations

The automated compliance verification framework represents a significant advancement over traditional manual compliance approaches, addressing the limitations of current healthcare data-sharing systems that rely on periodic auditing and reactive security incident responses [70]. The framework’s ability to provide real-time compliance verification and transparent audit mechanisms directly addresses the challenges outlined in the background analysis, where healthcare organizations struggle with the dynamic requirements of modern, multi-organizational data-sharing scenarios [71].

However, the complexity of healthcare privacy regulations necessitates a hybrid approach, as demonstrated in the proposed architecture, combining automated routine task processing with expert human oversight for contextual legal interpretation. This design acknowledges that while smart contracts can effectively automate routine compliance tasks such as consent verification and access logging, the nuanced understanding of medical necessity, privacy risk assessment, and determination of appropriate safeguards require human expertise that cannot be fully algorithmized [72].

The framework’s implementation of both HIPAA and GDPR requirements within a single architectural solution addresses the critical research gap identified in the introduction, where previous studies have focused on individual regulatory frameworks rather than comprehensive multi-jurisdictional compliance. Legal liability and regulatory compliance assurance remain organizational responsibilities, regardless of the capabilities of automated systems. However, the proposed framework provides healthcare organizations with substantially enhanced tools for demonstrating due diligence and maintaining continuous compliance verification. Real-time audit trails and immutable transaction records create a compliance documentation system that significantly exceeds the capabilities of traditional centralized approaches, directly addressing the regulatory complexity challenges in current healthcare environments.

### 5.3. Implementation Challenges and Opportunities

Healthcare system integration through HL7 FHIR standards achieves high compatibility with existing healthcare data formats, supporting relatively seamless integration with contemporary healthcare information systems [73]. However, the complexity of implementation requires specialized technical expertise and substantial organizational commitment.

The economic implications of blockchain implementation include infrastructure investment, specialized personnel training, and ongoing operational costs that accumulate over time and scale with the level of network participation [74]. These considerations may create barriers for smaller healthcare organizations, potentially exacerbating existing disparities in access to healthcare technology.

### 5.4. Future Research Directions

Future research should focus on addressing scalability challenges by developing healthcare-optimized blockchain architectures that meet transaction throughput requirements while maintaining security and compliance. Comprehensive real-world pilot studies in actual healthcare environments would validate theoretical findings under authentic operational conditions.

The most critical immediate direction is to validate the proposed framework empirically. This will involve building a functional prototype on Hyperledger Fabric and conducting comprehensive performance benchmarks using tools like Hyperledger Caliper to measure throughput, latency, and resource consumption under scalable loads, as outlined in Section 3.4.

Specific areas for future investigation include integrating machine learning algorithms to analyze blockchain audit trails for anomaly detection and to proactively identify potential security threats or compliance breaches in real time. Advanced cryptographic techniques, such as zero-knowledge proofs, can be explored to enable privacy-preserving data analytics while maintaining patient confidentiality requirements.

Additionally, research on economic models that support equitable access to blockchain-based healthcare data-sharing systems would address the potential disparities in technology adoption. Investigating hybrid consensus mechanisms designed explicitly for healthcare workflows can optimize the balance between security, performance, and energy efficiency. Finally, the development of standardized APIs and interoperability protocols will facilitate seamless integration with existing healthcare information systems across different vendor platforms.

## 6. Conclusions

This research investigation provides critical insights into the applicability of blockchain technology in healthcare data-sharing environments, while identifying specific technical, regulatory, and organizational considerations for practical implementation. The systematic analysis revealed that although blockchain technology offers compelling advantages in security, transparency, and decentralized control, implementation considerations must be carefully evaluated before deployment in healthcare environments.

The proposed framework demonstrates enhanced security through decentralized control mechanisms and cryptographic protection protocols compared to traditional centralized architectures. Smart contract-based compliance verification has the potential to automate routine regulatory tasks while maintaining the necessary human oversight for complex legal interpretation.

The regulatory compliance automation framework achieves notable improvements in processing routine compliance tasks while revealing the need for human expert intervention in complex legal interpretation scenarios. Robust cryptographic security mechanisms effectively address regulatory requirements, including the GDPR’s right to erasure, through patient-controlled cryptographic key management systems.

Architectural analysis suggests that blockchain-based healthcare data-sharing systems offer enhanced security and decentralized control compared to traditional healthcare data-sharing systems. However, implementation considerations—including organizational readiness, technical complexity, and economic implications—require careful evaluation. The framework offers promising solutions for automating regulatory compliance, while addressing the fundamental limitations of centralized healthcare information systems.

Future research should address these identified considerations by developing healthcare-optimized blockchain architectures, conducting comprehensive real-world pilot studies in healthcare environments, and integrating with emerging technologies to enhance system capabilities and accessibility.

## Figures and Tables

**Table 1:** Legal issues about extracting the pseudo-code.

Q001 Does the research study involve the examination of medical records?
Q002 Does the study generate new medical records?
Q003 Is the research authorized to use PHI with participant consent?
Q004 Does the research impact the privacy rights and well-being of individuals whose records will be used?
Q005 Can the research be practically conducted without obtaining a waiver?
Q006 Is it feasible to conduct the research without utilizing PHI?
Q007 Is there a reasonable balance between the privacy risks and the anticipated benefits of the research?
Q008 Does the research proposal include a suitable plan to promptly destroy identifiers or provide justification for their retention?
Q009 Is there a written assurance in the research documentation that PHI will not be reused or disclosed for other purposes?
Q010 Is the collection of patient contact information essential for conducting the research?
Q011 Do research participants have a right to access their research records?

**Table 2:** Pseudo-code: HIPAA authorization to use PHI.

Authorization Status	Required Initial Conditions	Required Path Condition (* Q003)	Other Required Conditions (* Q004–* Q011)
GRANTED (True)	(* Q001 == YES) OR (* Q002 == YES)	* Q003 == YES	N/A (Authorization is granted immediately)
GRANTED (True)	(* Q001 == YES) OR (* Q002 == YES)	* Q003 == NO	This path requires a HIPAA Waiver of Authorization, meaning ALL criteria below must be met: * Q004 (Impact on privacy) = NO * Q005 (Impracticable without waiver) = NO * Q006 (Feasible without PHI) = NO * Q007 (Risk/Benefit balance) = YES * Q008 (Plan to destroy identifiers) = YES * Q009 (No reuse assurance) = YES * Q010 (Contact essential) = YES * Q011 (Right to access records) = YES
DENIED (False)	(* Q001 == YES) OR (* Q002 == YES)	* Q003 == NO	ANY of the specific Q004–Q011 conditions above are NOT met.

The ”*” denotes the corresponding question in Table 1.

## Data Availability

No new data were created or analyzed in this study. Data sharing does not apply to this article.

## References

[1] Kruse C.S., Frederick B., Jacobson T., Monticone D.K. (2017). Cybersecurity in healthcare: A systematic review of modern threats and trends. Technol. Health Care.

[2] Sittig D.F., Singh H. (2010). A new sociotechnical model for studying health information technology in complex adaptive healthcare systems. BMJ Qual. Saf..

[3] Gordon W.J., Catalini C. (2018). Blockchain technology for healthcare: Facilitating the transition to patient-driven interoperability. Comput. Struct. Biotechnol. J..

[4] Kumar T., Ramani V., Ahmad I., Braeken A., Harjula E., Ylianttila M. Blockchain utilization in healthcare: Key requirements and challenges. Proceedings of the 2018 IEEE 20th International Conference on e-Health Networking, Applications and Services (Healthcom).

[5] Razdan S., Sharma S. (2022). Internet of medical things (IoMT): Overview, emerging technologies, and case studies. IETE Tech. Rev..

[6] Adler-Milstein J., Jha A.K. (2017). HITECH Act drove large gains in hospital electronic health record adoption. Health Aff..

[7] Blumenthal D., Tavenner M. (2010). The “meaningful use” regulation for electronic health records. N. Engl. J. Med..

[8] Kellermann A.L., Jones S.S. (2013). What it will take to achieve the as-yet-unfulfilled promises of health information technology. Health Aff..

[9] Esmaeilzadeh P. (2020). Use of AI-based tools for healthcare purposes: A survey study from consumers’ perspectives. BMC Med. Inform. Decis. Mak..

[10] Davis F.D., Bagozzi R.P., Warshaw P.R. (1989). User acceptance of computer technology: A comparison of two theoretical models. Manag. Sci..

[11] Haque A.B., Islam A.N., Hyrynsalmi S., Naqvi B., Smolander K. (2021). GDPR compliant blockchains–a systematic literature review. IEEE Access.

[12] Act A. (1996). Health insurance portability and accountability act of 1996. Public Law.

[13] Voigt P., Von dem Bussche A. (2017). The Eu General Data Protection Regulation (Gdpr): A Practical Guide.

[14] Nosowsky R., Giordano T.J. (2006). The Health Insurance Portability and Accountability Act of 1996 (HIPAA) privacy rule: Implications for clinical research. Annu. Rev. Med..

[15] McKinstry C.J. (2018). The HIPAA privacy rule: Flawed privacy exposed when compared with the European Union’s general data protection regulation. J. Health Care Financ..

[16] Elkourdi F., Wei C., Xiao L., YU Z., Asan O. (2024). Exploring current practices and challenges of HIPAA compliance in software engineering: Scoping review. IEEE Open J. Syst. Eng..

[17] Jeyaraman N., Ramasubramanian S., Yadav S., Balaji S., Muthu S., Jeyaraman M. (2024). Regulatory challenges and frameworks for fog computing in healthcare. Cureus.

[18] Susha I., Rukanova B., Zuiderwijk A., Gil-Garcia J.R., Hernandez M.G. (2023). Achieving voluntary data sharing in cross sector partnerships: Three partnership models. Inf. Organ..

[19] Khan S., Khan M., Khan M.A., Wang L., Wu K. (2025). Advancing medical innovation through blockchain-secured federated learning for smart health. IEEE J. Biomed. Health Inform..

[20] Oh J., Son S., Kwon D., Kim M., Park Y., Park Y. (2024). Design of secure and privacy-preserving data sharing scheme based on key aggregation and private set intersection in medical information system. Mathematics.

[21] Agbo C.C., Mahmoud Q.H., Eklund J.M. (2019). Blockchain technology in healthcare: A systematic review. Healthcare.

[22] Coventry L., Branley D. (2018). Cybersecurity in healthcare: A narrative review of trends, threats and ways forward. Maturitas.

[23] Chen L., Lee W.-K., Chang C.-C., Choo K.-K.R., Zhang N. (2019). Blockchain based searchable encryption for electronic health record sharing. Future Gener. Comput. Syst..

[24] Zhang P., White J., Schmidt D.C., Lenz G., Rosenbloom S.T. (2018). FHIRChain: Applying blockchain to securely and scalably share clinical data. Comput. Struct. Biotechnol. J..

[25] Bender D., Sartipi K. HL7 FHIR: An Agile and RESTful approach to healthcare information exchange. Proceedings of the 26th IEEE International Symposium on Computer-Based Medical Systems.

[26] Mandl K.D., Markwell D., MacDonald R., Szolovits P., Kohane I.S. (2001). Public standards and patients’ control: How to keep electronic medical records accessible but private Medical information: Access and privacy Doctrines for developing electronic medical records Desirable characteristics of electronic medical records Challenges and limitations for electronic medical records Conclusions Commentary: Open approaches to electronic patient records Commentary: A patient’s viewpoint. BMJ.

[27] Agrawal T.K., Kumar V., Pal R., Wang L., Chen Y. (2021). Blockchain-based framework for supply chain traceability: A case example of textile and clothing industry. Comput. Ind. Eng..

[28] Paulk M.C., Curtis B., Chrissis M.B., Weber C.V. (1993). Capability Maturity Model.

[29] Nakamoto S. (2008). Bitcoin: A peer-to-peer electronic cash system. Available SSRN 3440802.

[30] Hawlitschek F., Notheisen B., Teubner T. (2020). A 2020 perspective on “The limits of trust-free systems: A literature review on blockchain technology and trust in the sharing economy”. Electron. Commer. Res. Appl..

[31] Auinger A., Riedl R. (2018). Blockchain and Trust: Refuting Some Widely-Held Misconceptions. https://scholar.archive.org/work/n6zr4u7yqrd7deznqz7y5bcfga/access/wayback/https://aisel.aisnet.org/cgi/viewcontent.cgi?article=1246&context=icis2018.

[32] Werbach K. (2018). Trust, but verify: Why the blockchain needs the law. Berkeley Technol. Law J..

[33] Stach C., Gritti C., Przytarski D., Mitschang B. (2022). Assessment and treatment of privacy issues in blockchain systems. ACM SIGAPP Appl. Comput. Rev..

[34] Ghesmati S., Fdhila W., Weippl E. User-perceived privacy in blockchain. Proceedings of the International Conference on Financial Cryptography and Data Security.

[35] Marthews A., Tucker C. (2023). What blockchain can and can’t do: Applications to marketing and privacy. Int. J. Res. Mark..

[36] Zhang J., Yang Y., Liu X., Ma J. (2022). An efficient blockchain-based hierarchical data sharing for healthcare internet of things. IEEE Trans. Ind. Inform..

[37] Stefanescu D., Montalvillo L., Galán-García P., Unzilla J., Urbieta A. (2022). A systematic literature review of lightweight blockchain for IoT. IEEE Access.

[38] Harshini Poojaa K., Ganesh Kumar S. (2022). Scalability challenges and solutions in blockchain technology. Inventive Computation and Information Technologies: Proceedings of ICICIT 2021.

[39] Nasir M.H., Arshad J., Khan M.M., Fatima M., Salah K., Jayaraman R. (2022). Scalable blockchains—A systematic review. Future Gener. Comput. Syst..

[40] Pradhan N.R., Singh A.P., Verma S., Kavita, Wozniak M., Shafi J., Ijaz M.F. (2022). Author Correction: A blockchain based lightweight peer-to-peer energy trading framework for secured high throughput micro-transactions. Sci. Rep..

[41] Qiu X., Chen W., Tang B., Liang J., Dai H.-N., Zheng Z. (2022). A distributed and privacy-aware high-throughput transaction scheduling approach for scaling blockchain. IEEE Trans. Dependable Secur. Comput..

[42] Wu H., Liu H., Li J. (2023). FabricETP: A high-throughput blockchain optimization solution for resolving concurrent conflicting transactions. Peer-Peer Netw. Appl..

[43] Barbaria S., Mahjoubi H., Rahmouni H.B. (2023). A novel blockchain-based architectural modal for healthcare data integrity: Covid19 screening laboratory use-case. Procedia Comput. Sci..

[44] Chakraborty S., Kadri S. (2022). Utilisation of Blockchain Technology for Better Health Outcomes during COVID-19. New Frontiers in Communication and Intelligent Systems.

[45] Pal S., Dorri A., Jurdak R. (2022). Blockchain for IoT access control: Recent trends and future research directions. J. Netw. Comput. Appl..

[46] Roosan D., Wu Y., Tatla V., Li Y., Kugler A., Chok J., Roosan M.R. (2022). Framework to enable pharmacist access to health care data using Blockchain technology and artificial intelligence. J. Am. Pharm. Assoc..

[47] Omar I.A., Jayaraman R., Salah K., Simsekler M.C.E., Yaqoob I., Ellahham S. (2020). Ensuring protocol compliance and data transparency in clinical trials using Blockchain smart contracts. BMC Med. Res. Methodol..

[48] Boussi Rahmouni H., Munir K. (2021). An ontology-based compliance audit framework for medical data sharing across Europe. Int. Arab J. Inf. Technol. (IAJIT).

[49] Vegoda P.R. (1987). Introduction to hospital information systems. Int. J. Clin. Monit. Comput..

[50] Sushma K., Viji C., Rajkumar N., Ravi J., Stalin M., Najmusher H. (2023). Healthcare 4.0: A review of phishing attacks in cyber security. Procedia Comput. Sci..

[51] Kumar M., Raj H., Chaurasia N., Gill S.S. (2023). Blockchain inspired secure and reliable data exchange architecture for cyber-physical healthcare system 4.0. Internet Things Cyber-Phys. Syst..

[52] Khatri S., Alzahrani F.A., Ansari M.T.J., Agrawal A., Kumar R., Khan R.A. (2021). A systematic analysis on blockchain integration with healthcare domain: Scope and challenges. IEEE Access.

[53] Khatoon A. (2020). A blockchain-based smart contract system for healthcare management. Electronics.

[54] Sharma A., Sarishma, Tomar R., Chilamkurti N., Kim B.-G. (2020). Blockchain based smart contracts for internet of medical things in e-healthcare. Electronics.

[55] Regulation P. (2018). General data protection regulation. Intouch.

[56] Bakare S.S., Adeniyi A.O., Akpuokwe C.U., Eneh N.E. (2024). Data privacy laws and compliance: A comparative review of the EU GDPR and USA regulations. Comput. Sci. IT Res. J..

[57] Kammueller F. Formal modeling and analysis of data protection for GDPR compliance of IoT healthcare systems. Proceedings of the 2018 IEEE International Conference on Systems, Man, and Cybernetics (SMC).

[58] Wang Q., Qin S. (2021). A hyperledger fabric-based system framework for healthcare data management. Appl. Sci..

[59] Solaiman E., Wike T., Sfyrakis I. (2021). Implementation and evaluation of smart contracts using a hybrid on- and off-blockchain architecture. Concurr. Comput. Pract. Exp..

[60] He F., Li F., Liang P. (2024). Enhancing smart contract security: Leveraging pre-trained language models for advanced vulnerability detection. IET Blockchain.

[61] Griggs K.N., Ossipova O., Kohlios C.P., Baccarini A.N., Howson E.A., Hayajneh T. (2018). Healthcare blockchain system using smart contracts for secure automated remote patient monitoring. J. Med. Syst..

[62] Chowdhary A., Huang D., Mahendran J.S., Romo D., Deng Y., Sabur A. Autonomous security analysis and penetration testing. Proceedings of the 2020 16th International Conference on Mobility, Sensing and Networking (MSN).

[63] Jones J., Gottlieb D., Mandel J.C., Ignatov V., Ellis A., Kubick W., Mandl K.D. (2021). A landscape survey of planned SMART/HL7 bulk FHIR data access API implementations and tools. J. Am. Med. Inform. Assoc..

[64] Ravuri A., Sendil M.S., Rani M., Srikanth A., Sharath M., Sudarsa D., Gupta K.G. Blockchain-enabled collaborative anomaly detection for IoT security. Proceedings of the MATEC Web of Conferences.

[65] Sukhwani H., Wang N., Trivedi K.S., Rindos A. Performance modeling of hyperledger fabric (permissioned blockchain network). Proceedings of the 2018 IEEE 17th International Symposium on Network Computing and Applications (NCA).

[66] Ante L., Saggu A. (2024). Time-varying bidirectional causal relationships between transaction fees and economic activity of subsystems utilizing the ethereum blockchain network. J. Risk Financ. Manag..

[67] Guggenberger T., Sedlmeir J., Fridgen G., Luckow A. (2022). An in-depth investigation of the performance characteristics of Hyperledger Fabric. Comput. Ind. Eng..

[68] De Aguiar E.J., Faiçal B.S., Krishnamachari B., Ueyama J. (2020). A survey of blockchain-based strategies for healthcare. ACm Comput. Surv. (CsUr).

[69] Baboi M. (2023). Security of consensus mechanisms in blockchain. Rom. Cyber Secur. J..

[70] Haritha T., Anitha A. (2023). Multi-level security in healthcare by integrating lattice-based access control and blockchain-based smart contracts system. IEEE Access.

[71] Odeh A., Abdelfattah E., Salameh W. (2024). Privacy-preserving data sharing in telehealth services. Appl. Sci..

[72] McGregor L., Murray D., Ng V. (2019). International human rights law as a framework for algorithmic accountability. Int. Comp. Law Q..

[73] Duda S.N., Kennedy N., Conway D., Cheng A.C., Nguyen V., Zayas-Cabán T., Harris P.A. (2022). HL7 FHIR-based tools and initiatives to support clinical research: A scoping review. J. Am. Med. Inform. Assoc..

[74] Gökalp E., Gökalp M.O., Çoban S., Eren P.E. (2018). Analysing opportunities and challenges of integrated blockchain technologies in healthcare. Eurosymp. Syst. Anal. Des..

